# A Neonatal Thrombosis Patient Treated Successfully with Recombinant Tissue Plasminogen Activator

**DOI:** 10.4274/Tjh.07641

**Published:** 2013-09-05

**Authors:** Kemal Erdinç, Serdar Ümit Sarıcı, Orçun Dabak, Orhan Gürsel, Adem Güler, Ahmet Emin Kürekçi, Fuat Emre Canpolat

**Affiliations:** 1 Gülhane Military Academy of Medicine, Department of Pediatrics, Ankara, Turkey; 2 Gülhane Military Academy of Medicine, Departments of Pediatrics and Neonatology, Ankara, Turkey; 3 Gülhane Military Academy of Medicine, Departments of Pediatrics and Hematology, Ankara, Turkey; 4 Gülhane Military Academy of Medicine, Department of Cardiovascular Surgery, Ankara, Turkey

**Keywords:** Thrombosis, Neonate, Tissue plasminogen activator, Preterm

## Abstract

Herein we report an asphyctic preterm neonate with respiratory distress and prothrombotic risk factors that responded positively to rtPA treatment following 2 attacks of acute thrombosis.

**Conflict of interest:**None declared.

## Abstract (tr)

Bu yazıda solunum sıkıntısı ve asfiksi ile doğmuş, protrombotik risk faktörleri de olan bir prematüre bebekte iki tromboz atağının rekombinant doku plazminojen aktivatörü ile başarılı bir şekilde tedavi edildiği sunulmuş, ilacın etkinliği ve bu grup hastalarda dahi uygulanılabilirliği vurgulanmıştır.

## INTRODUCTION

Thrombolytic treatment has increased in importance in recent years due to a significant increase in the number of neonatal cases diagnosed with thrombosis as a result of diagnostic and therapeutic interventions performed during the neonatal period [1]. Thrombolytic treatment is an alternative treatment for the elimination of thrombi, which may cause life-threatening organ and tissue destruction. Recombinant tissue plasminogen activator (rtPA), which is commonly used for the treatment of myocardial infarction and stroke in adults, has also been used to treat cardiac thrombi in patients with congenital heart disease and right-to-left shunting [2,3,4]. Research on the use of rtPA in neonates is limited [5]. Herein we report an asphyctic preterm neonate with respiratory distress and prothrombotic risk factors that responded positively to rtPA treatment following 2 attacks of acute thrombosis. 

## CASE REPORT

A preterm male neonate weighing 940 g that was born to a 25-year-old gravida 8, para 2 mother was transferred 3h after birth due to respiratory distress from another hospital to our neonatal intensive care unit in an intubated and hand (bag)-ventilated manner in a transport incubator. The mother had not menstruated during the previous 2 years and, therefore, the neonate’s gestational age was estimated to be 27 weeks based on the Ballard score. The mother’s obstetric history included 5 abortions and 2 curettages, and an emergency cesarean section due to placenta previa the day she presented with vaginal bleeding to a hospital in a neighboring town. 

Physical examination of the neonate showed hypothermia, hypotonia, cyanosis, intercostal and subcostal retractions, grunting, and tachypnea. The patient was diagnosed with hyaline membrane disease, and mechanical ventilation with intratracheal surfactant treatment, parenteral fluid, and empirical antibiotic treatment was initiated. Coldness, pallor, and cyanosis developed in the distal region of the right forearm soon after venipuncture of the right antecubital vein. Pulse oximetry showed no impulse (pulse and saturation) from this region, distal pulses could not be palpated, and Doppler ultrasonography showed positive brachial pulses, and negative radial and ulnar pulses. 

The thrombosis was located at the distal end of the right brachial artery. There was no blood passing to the distal region. Hematochezia was observed 8h after birth. Intravenous alteplase infusion (0.2 mg·kg·h–1) was initiated as treatment for the thrombosis in the right forearm. Prior to the initiation of this treatment basal hematologic factor levels and etiologic hematologic parameters were as follows: D-dimer: 12 ng mL–1; aPTT: 45 s; PT: 13 s; INR: 176 mg dL–1; protein C activity: 21%; protein S activity: 34%; AT III level: 22 mg dL–1; platelet count: 231,000 mm–3. Infusion was stopped after 30 min, following complete recovery. Hematochezia was subsequently observed 3 times during the 1st d of life, but did not recur during the 2nd day of life. 

The patient was extubated after 4 day of mechanical ventilation support. On the 13th d of life spontaneous and acute thrombosis recurred in the distal region of the left foot, and coldness, pallor, and cyanosis developed in the 1st, 2nd, and 4th phalanges of the left foot. Blood was withdrawn to analyze the coagulation profile, and heparin treatment with a 100 U kg–1 loading dose and 25 U·kg·h–1 maintenance dose, and intravenous alteplase infusion (0.2 mg·kg·h–1) were initiated and administered for 8 h. Fresh frozen plasma infusion (10 mL kg–1 b.i.d.) was also initiated. This treatment regimen resulted in complete recovery in 48 h. Etiologic investigation of the recurring thrombus formation showed that the neonate had a heterozygous factor V Leiden mutation, a homozygous plasminogen activator inhibitor-1 (PAI-1) 5G/5G polymorphism, a heterozygous methyl tetrahydrofolate reductase (MTHFR) polymorphism, and a normal prothrombin 20210A gene structure. Maintenance treatment with subcutaneous low-molecular weight heparin (enoxaparin) (0.2 mg kg–1 b.i.d.) was initiated and a therapeutic anti-factor Xa level of 0.5-1 U mL–1 was targeted. At the time this report was written, the patient was being followed-up as an outpatient with the diagnosis of chronic lung disease of prematurity.

## DISCUSSION

The incidence of symptomatic neonatal thrombosis, including that in the central nervous system, is reported to be 0.51 per 10,000 live births [1]. Along with advancements in diagnosis and treatment, and because of various invasive interventions performed in neonates, the annual incidence may increase to 2.4 per 1000 in neonatal intensive care units [6]. Thrombi may occur at arterial catheter entry regions and in the presence of prothrombotic risk factors, such as factor V Leiden mutation, and deficiencies in protein C and protein S.

There is no consensus concerning the optimal mode of treatment for thrombosis, especially in neonates. Thrombolytic treatment is being used with increasing frequency for thrombi in neonates. Thrombolytic treatment is an alternative treatment for the elimination of thrombi, which can cause life-threatening organ and tissue destruction [7]. 

A study that included 14 neonate with a diagnosis of thrombosis treated with rtPA reported that is was a safe treatment option [8]. rtPA has been effectively used to treat catheter-related intracardiac thrombus and vena cava inferior thrombus [9,10]. A study that included 20 children (age between 1 day and 16 years) with thrombosis reported that 11 patients treated with rtPA had a complete response [11]. In the presented case, the mother’s history of recurrent abortus, hematochezia during the first hours of life, and development of an acute thrombus in the region of the brachial artery suggested that the patient had an underlying prothrombotic disease. In addition to this clinical finding as the patient was born preterm (estimated gestational age: 27 weeks) and had respiratory distress syndrome we chose to treat the infant with rtPA in accordance with a previous report of a patient with a brachial artery thrombus that was successfully treated with rtPA [12]. 

rtPA is characterized by a locally potent effect, which helps minimize the occurrence of systemic side effects. rtPA has a rapid curative effect on thrombi, which is a great benefit as it can minimize the incidence of acute tissue and organ necrosis. In the presented case a rapid response to the patient’s first attack of brachial artery thrombus was achieved using rtPA. Although heparin was required in addition to rtPA for the patient’s second attack of thrombus (lower extremity), complete recovery was achieved, indicating that the combination of these 2 drugs is safe for the treatment of recurrent thrombi. The combination of heparin and thrombolytic treatment has previously been reported to be safe and effective [13]. Although data about the use of this drug combination in neonates, especially preterm neonates, are limited, it is an alternative choice of treatment in cases of life-threatening illness and for preventing acute necrosis in extremities due to thrombus [5]. The presented case had a complete response without any tissue loss, following rapid diagnosis and treatment. The literature contains few reports on the use of rtPA in preterm neonates, and fewer cases with birth weights <1000 g, as in the presented case [14]. The presented case shows that use of rtPA in preterm neonates, even repeated doses, can be safe and effective; however, larger prospective, controlled clinical trials are needed to more fully demonstrate the safety and effectiveness of rtPA in neonates. We have written inform consent.

## ACKNOWLEDGEMENT

We are grateful to Nejat Akar, MD and Yonca Eğin, PhD, of Ankara University, School of Medicine, Department of Molecular Hematology. 

## CONFLICT OF INTEREST STATEMENT

The authors of this paper have no conflicts of interest, including specific financial interests, relationships, and/ or affiliations relevant to the subject matter or materials included.
